# Explainable Artificial Intelligence in Endocrinological Medical Research

**DOI:** 10.1210/clinem/dgab237

**Published:** 2021-05-21

**Authors:** Bobbie-Jo M Webb-Robertson

**Affiliations:** 1 Biological Sciences Division, Pacific Northwest National Laboratory, Richland, WA, USA; 2 Department of Biostatistics and Informatics, University of Colorado Anschutz Medical Campus, Aurora, CO, USA; 3 Department of Pathology, Immunology and Laboratory Medicine, University of Florida, Gainesville, FL, USA; 4 Department Biomedical Engineering, Oregon Health & Science University, Portland, OR, USA

**Keywords:** Artificial intelligence, height prediction, random forest, child growth, growth analyses

Artificial Intelligence (AI) has been a part of the medical community for decades in the form of Clinical Decision Support Systems to aid physicians in diagnosis and categorization of patients (1). Recent years have seen a shift from expert-derived models to proposed machine learning (ML) models into Clinical Decision Support Systems due to the ability of ML models to make predictions more accurately by exploiting higher dimensional and often complex data. In many cases ML models gain their advantage in accuracy by capturing complex and often nonlinear relationships between features being used to make the prediction. However, the hype and excitement around these methods are tempered by the limited utility of often black-box solutions in a clinical setting. This is driven by skepticism of results that are difficult for practitioners to not only interpret but explain to their patients (1-3). This skepticism is not unfounded as multiple examples of black-box solutions identifying incidental correlates as the key predictors have highlighted the potential bias in a training set, or reward system, that a ML model may exploit; an example of this is a model discerning wolves from huskies based on snow in the background rather than features of the dogs (1, 4, 5).

AI and ML is a growing field for endocrinology (6) and has already made inroads in the treatment of diabetes (7). Endocrinology is especially well positioned to take advantage of the upsurge in ML work focused on interpretability and explainability, which focus on developing models based on the trade-off between model accuracy and transparency rather than accuracy alone (5). In their recent article in *The Journal of Clinical Endocrinology & Metabolism,* Shmoish et al. (8), demonstrate the power of an explainable ML model to predict adult height from growth measurements of children before and at the age of 6 years. In this work they not only demonstrate superior performance in the context of accuracy but utilize the explainable model to provide insight into the factors underlying that performance as well as understand the potential deficiencies in the model that could be addressed in future developments.

The prediction of adult height was undertaken via a suite of ML algorithms and compared with 3 common expert-derived metrics: target height, conditional target height, and “grandma” method. Overall, there was not a lot of difference between the 3 expert-derived methods, the first 2 approaches that use parental height vs the last that is a doubling of baby length at a specified age. However, in all cases the ML model dramatically improves overall prediction of adult height.

Generalization is an important component of ML models, which means that the model can adapt properly to previously unseen data. Utilizing data that has been collected independently from the training data, but expected to be drawn from the same distribution, is one of the best strategies for this (5). Shmoish et al. (8), demonstrate that their model is robust to both where and how the measurements are collected. The results of the model, which showed a Pearson correlation between the predicted and observed adult height of R = 0.87 in the validation set associated with the training data (Swedish cohort of children born in 1974), was approximately the same, R = 0.88, in another Swedish cohort started in 1990 and a separate cohort in Edinburgh with children born between 1972 and 1976, R = 0.88.

The explainability of the model is based on feature importance metrics that can be directly extracted from the ML model of choice, a Random Forest, which is a benefit of the Random Forest ML approach in addition to the often high prediction accuracy. The most important features driving the model were the average height of the child between the age of 3.4-4 years of age and sex, with secondary features such as growth velocity and weight not playing an important role in the prediction. The authors then explored the model results to identify potential biases and infer reasons for these based on their expertise in the field. One interesting example is that the model is more accurate for girls than boys with a possible tie back to the time that girls reach adult height vs boys, which may indicate that 6 years of age is too young for a highly accurate prediction for boys. There also appears to be a shift in prediction towards the mean, meaning the short and tall subjects are either over- or under-estimated, respectively, which may be due to either the Random Forest approach or potentially a lack of adequate data at the extremes. These insights, as well as inclusion of potential environmental impacts on growth, offer a strong foundational model for future researchers to build upon.

In conclusion, Shmoish et al. (8), present a ML model to predict adult height based on growth measurements easily attained in any clinical setting demonstrating accurate predictions based solely on observational data. Furthermore, they delve into the model to identify the most important features, which helps to both understand why the model performs so well on validation and independent data and why it may be challenged in specific groups of the population, such as extremely short or tall individuals. These types of explainable AI models are well suited to use easily acquired patient data in the clinic and thus have the potential to transform how endocrine disorders are diagnosed and treated.

## Data Availability

Not applicable as there were not datasets generated or analyzed.

## Abbreviations

AI: artificial intelligence
ML: machine learning

## Additional Information

***Disclosures***: The author has nothing to disclose.
